# Artificial Intelligence With Robotics in Healthcare: A Narrative Review of Its Viability in India

**DOI:** 10.7759/cureus.39416

**Published:** 2023-05-23

**Authors:** Niyati Deo, Ashish Anjankar

**Affiliations:** 1 Medical School, Jawaharlal Nehru Medical College, Datta Meghe Institute of Medical Sciences, Wardha, IND; 2 Biochemistry, Jawaharlal Nehru Medical College, Datta Meghe Institute of Medical Sciences, Wardha, IND

**Keywords:** logistics, development, healthcare, artificial intelligence, robotics

## Abstract

This short review focuses on the emerging role of artificial intelligence (AI) with robotics in the healthcare sector. It may have particular utility for India, which has limited access to healthcare providers for a large growing population and limited health resources in rural India. AI works with an amalgamation of enormous amounts of data using fast and complex algorithms. This permits the software to quickly adapt the pattern of the data characteristics. It has the possibility to collide with most of the facets of the health system which may range from discovery to prediction and deterrence. The use of AI with robotics in the healthcare sector has shown a remarkable rising trend in the past few years. Functions like assistance with surgery, streamlining hospital logistics, and conducting routine checkups are some of the tasks that may be managed with great efficiency using artificial intelligence in urban and rural hospitals across the country. AI in the healthcare sector is advantageous in terms of ensuring exclusive patient care, safe working conditions where healthcare providers are at a lower risk of getting infected, and perfectly organized operational tasks. As the healthcare segment is globally recognized as one of the most dynamic and biggest industries, it tends to expedite development through modernization and original approaches. The future of this lucrative industry is looking forward to a great revolution aiming to create intelligent machines that work and respond like human beings. The future perspective of AI and robotics in the healthcare sector encompasses the care of elderly people, drug discovery, diagnosis of deadly diseases, a boost in clinical trials, remote patient monitoring, prediction of epidemic outbreaks, etc. However, the viability of using robotics in healthcare may be questionable in terms of expenditure, skilled workforce, and the conventional mindset of people. The biggest challenge is the replication of these technologies to the smaller towns and rural areas so that these facilities may reach the larger segment of the entire population of the country. This review aims to examine the adaptability and viability of these new technologies in the Indian scenario and identify the major challenges.

## Introduction and background

The status of the healthcare sector in India is far from providing universal healthcare coverage to the entire population and lags behind many developing and few least developed countries in terms of health indicators. In addition to this, there are large disparities among various states in achieving the desired health outcomes, as well as the establishment of a sound information system. The adoption of the National Health Policy of India in 2017 has largely facilitated the bridging of the gap among various stakeholders of National Healthcare through the digital corridor. The policy recognizes the significant role of technology in healthcare delivery. It advocates the setting up of a National Digital health authority (NDHA) to regulate, develop and deploy digital health within the field of care. National Institution for Transforming India (NITI) Aayog, after being authorized by the Government of India to draft a National Strategy on Artificial Intelligence (AI) emphasized five sectors that would benefit the most from AI in 2018, of which healthcare is one [1]. 

The application of AI in healthcare may be classified into four broad categories, i.e. expressive, analytical, prognostic, and prescriptive. The gap created by a lack of skilled healthcare professionals can only be bridged by enhancing the use of AI in the health sector. Usual health issues can easily be diagnosed with the help of AI, thus reducing the workload of expert health professionals as well as reducing the cost of treatment in India [2]. It is envisaged that by the year 2035, AI would be able to enhance the economy of India by adding 957 billion USD to it (Accenture, 2017) [2]. AI will also prove to be a medium for reducing the economic disparity in the country. A report of the TCS global survey (TCS, 2017) projects that the visible reduction of jobs by AI could possibly be replaced by the creation of new jobs in the upcoming AI-integrated healthcare projects [2].

As a matter of fact, the healthcare setup in India is not perfect. It is deficient in terms of the availability of doctors, nurses, medical technicians, and healthcare facilities needed to attend to the community. The number of qualified doctors is insufficient for the rapidly growing needs of the Indian healthcare system. At the same time, these doctors are concentrated in urban areas and there is a huge gap in medical personnel in rural areas as compared to urban settings. Approximately 74% of the graduate doctors in India work in urban areas which cater to only about one-fourth of the population [3]. Because of the maldistribution of resources, each doctor serves 19,000 people [4]. India will need 2.3 million doctors by 2030 to reach the minimum doctor-patient ratio of 1:1000, which the World Health Organization recommends. The early ideas by a few dozen of healthcare startups have the potential to boost the Indian healthcare systems in the future and also have the capability to reduce the burden of the healthcare system.

Recently the coronavirus disease 2019 (COVID-19) pandemic posed a great challenge to the healthcare sector creating a huge demand for equipment, medicines AI-based applications, and robotics. Many reputed hospitals all over the world have switched over to AI and robotic procedures during the COVID-19 pandemic for functions like disinfection and screening of patients and employees at the entry point. Measures such as distantly supervised surgeries, distance education, telemedicine, and video conferencing with doctors were used during the recent pandemic. The experience gained during the pandemic has primarily enhanced the adaptability for use of robotics in the healthcare sector [5].

AI's major forms of relevance in healthcare are as follows: 1. Machine learning: The use and development of complete systems that are able to learn and adapt without explicit instructions to analyze and draw inferences from data patterns; 2. Natural language processing: A specialized branch of AI focused on the interpretation and manipulation of human-generated written or spoken data; 3. Robotic process automation: An automation technology that uses software to mimic the back office tasks of human workers, such as extracting data, filling the forms, moving files, etc.

In addition, AI also supports the healthcare system in diagnosis and treatment applications, patient engagement and adherence, and administrative applications [6]. AI not only simplifies the work of doctors, nurses, and other healthcare workers but also saves an ample amount of time. Thus the adoption of digital solutions for the prevention, diagnosis, and cure of various ailments is the wise route for India to deal with the aim of providing health for all. 

## Review

Research methodology

The present study was conducted between the months of April to June 2022. Databases like Pubmed and Google Scholar were mainly used to search the literature. Databases like Scopus and Web of Science were excluded. Most of the research publications taken into account for gathering the data were from 2013 to 2022. Research papers related to the use of robotics and artificial intelligence in healthcare were thoroughly studied with special emphasis on its viability in the Indian scenario. The relevant search terms used were artificial intelligence, robotics, healthcare, India, etc. It was a difficult task to explore the required information, as meager data is available regarding the use of robotics in the Indian healthcare sector which requires enhanced attention of researchers. 

Functioning of robotics in healthcare

Working of robotics in healthcare comprises AI applications like machine learning and deep learning. AI works with an amalgamation of vast amounts of data using fast and intelligent algorithms. This permits the software to quickly adapt the pattern of the data characteristics. Execution of AI is basically program oriented and the designed program consists of the basic information as to how it has to work. All the data is fed into web platforms such as the “cloud” which have the potential to store massive data and information to be used through the internet. There are immense possibilities for development in the healthcare sector through the use of AI in the future [7].

The main objective of AI is to solve problems by gathering and analyzing the information provided by the program and sensors. Another goal is to learn and respond in uncommon situations by taking alternate ways and remembering the successful alternative to be used in similar situations. It works for creating proficient arrangements so that it can learn, think, and suggest the best possible ways to the users. They work towards accomplishing intellect in machines so that they can perform just like human beings [8].

**Figure 1:**
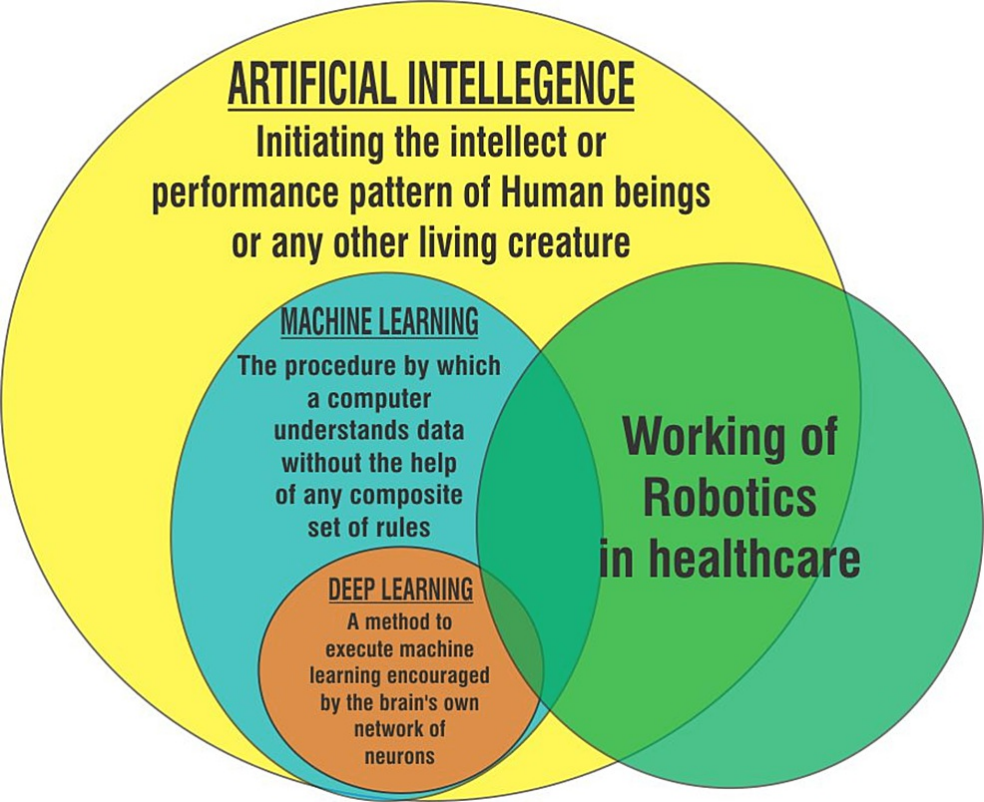
Working of Robotics in Healthcare. Image credit: Niyati Deo

Artificial intelligence has the possibility to collide with most of the facets of the health system which may range from discovery to forecast and deterrence. Although the rate of adherence to the new technologies is much lower than their appearance, it is needed that all healthcare professionals be trained uniformly to adopt these new technologies which include techniques like robotic process automation, natural language processing, machine learning, etc. [9]. The interplay between artificial intelligence, machine learning, and deep learning ultimately leads to the working of robotics in healthcare, which can be seen in Figure 1.

Use of robotics in healthcare

Assistance in Surgery

The application of robotics in surgery was first imagined in 1967, but it was just a dream for about 30 years until the United States defense department set up research organizations that gradually developed the first surgical robot designed to conduct different types of tasks. Initially, these robots were used during wars on the battlefields [10]. 

Today the most rapidly growing field with the application of robotics in healthcare is surgery. It aims to enhance the capabilities of humans and overcome human limitations in the field of surgery [11]. In India, the first urologic robot named da Vinci S was set up at the All India Institute of Medical Sciences, New Delhi in 2006. This initiation was followed by an exceptional expansion of robotic surgery in the country. Till July 2019 there were 66 centers and more than 500 skilled robotic surgeons in India who had successfully performed more than 12,800 surgeries with the assistance of robots [12]. This unexpected expansion of robotic surgery shows that the future of robotic surgery in India is very bright. The introduction of the da Vinci Surgical System is one of the biggest inventions in surgery [8]. The use of high-definition computer vision enables surgeons to get detailed information about the inner condition of the patients which enhances their performance during the surgery [13].

For many years engineers and medical researchers, are constantly trying to invent ways in which robotics can be used in surgery, as it has advantages like mechanical accuracy, permanence, and the ability to work in unsafe surroundings [14]. In the past few years, surgeries assisted by robots have played a significant role in boosting the Indian healthcare system. Reports show that hundreds of robotic surgeons are positioned at different hospitals in India. Surgeries performed with the help of robotics are thought to be better in comparison to other conventional methods due to their precision, shorter recovery periods, lesser pain, and blood loss. These kinds of surgeries are also preferred because they save traveling and boarding costs [15].

Robotic surgery has successfully sorted the limitations of laparoscopic surgery which is a big leap toward surgery with minimal access. As it may be predicted that almost all surgeries will be performed with robotic assistance in the future, a realistic training approach will be required to enhance the skills of surgeons, thus reshaping the knowledge curvature of the trainees by exposure to new methods like robotic surgical simulators and robotic telementoring [16]. The role of robotics is increasingly becoming crucial in surgeon training. For example, virtual reality simulators provide realistic situations and real training experiences to the trainees. Practicing the procedures becomes easy within the virtual environment [17].

Surgical robots are widely being used in over a million surgical actions related to various departments of the healthcare sector. AI helps the surgeon to get actual warnings and suggest appropriately during the process. Profound learning data helps a lot to provide the best surgical application suitable for the patient [18]. Robotics is also helpful in facilitating experts who are often concentrated in big cities and are not available for patients residing in small towns and rural areas. 

Support to Healthcare Workers

In addition to assistance in the operating room, robotics are also useful in clinics and Outdoor Patient Departments to enhance patient care. For example, robots were used to screen suspected patients at the entrance of health facilities during the COVID-19 pandemic. The use of automation and robots can also be seen in research laboratories where they are used to conduct many manual and repetitive tasks so that scientists can focus on more deliberate tasks and move faster towards discoveries. Remedial treatment after strokes, paralysis, traumatic brain injuries, etc. can be ensured with the help of therapeutic robots. These robots can monitor the patients as they perform prescribed exercises, and measure degrees of motion in various positions in a better way compared to the human eye. Social robots can also be used to interact with patients and also encourage them [19].

Logistic Arrangements

Medical robots efficiently streamline workflows and reduce risk which makes them more feasible to be used for many purposes. For example, robots can clean and organize patients' rooms autonomously, thus lowering the risk of interpersonal contact in infectious disease wards. Thus, for cleaning purposes, human support robots (HSR) are used [20]. Enabled medicine identifier software in robots helps in the distribution of medicines to patients in hospitals. Due to this kind of support hospital staff can devote more time to giving direct care to the patients.

Advantages of using robotics in healthcare

Exclusive Patient Care

Socially assistive robots (SARs) are the result of the development of AI along with physically assisted technologies. SARs are emotionally intelligent machines that lead to exclusive patient care, as these are capable of communicating with patients through a communicative range that makes them respond emotionally. The different types of response include interaction, communication, companionship, and emotional attachment [12]. Judicious use of robotics in the healthcare system ensures excellent patient care, perfect processes in medical surroundings, and a secure atmosphere for patients and medical professionals. Chances of human error and negligence are meager with the use of automated robots in healthcare. The health and social care sector is redefined by the invention and continuous development of SARs [12].

Protected Working Conditions

The role of nurses, ward boys, receptionists, and other healthcare workers can be easily performed by robots. The different types of robots: (i) receptionist robots, (ii) medical servers, (iii) nurse robots, etc., are capable of performing the above-mentioned roles very efficiently [15]. Automated mobile robots (AMRs) are used in many health facilities such as to distribute medical supplies and linen, collect data and information about patients, and serve food and water to patients in hospitals in order to keep medical professionals safe from pathogen exposure and thus prevent the spread of infections. Therefore, these robots were vigorously used during the recent COVID-19 pandemic. According to Podpora et al., hospitality robots like Wegree and Pepper developed by SoftBank Robotics in Japan were the most used robots during the pandemic, as they were helpful to control the rate of spreading of disease [15]. During the COVID-19 pandemic, excellent work was done for pandemic preparedness, screening, contact tracing, disinfecting, and enforcing quarantine and social distancing. The Arogya Setu app which was developed by National Informatics Centre and Information Technology Ministry has proven to be a boon in the management of the COVID-19 pandemic. Social robots are used for doing strenuous work like lifting heavy beds or transferring patients, thus reducing the physical strain on healthcare workers.

 Organized Operational Tasks

Automated mobile robots (AMRs) regularize regular tasks, decrease the physical burden on health workers, and make sure that more precise procedures are used. These robots can address the shortage of staff, keep a trail of records and place orders on time. They ensure that medicines and other equipment are available as and when needed. Rooms can be quickly cleaned and sanitized and are timely ready for incoming patients by automated robots which enable health professionals to perform other important patient-related jobs. Robots can be efficiently used for making diagnoses of different diseases by using artificial intelligence. The radiologist robots, which are equipped with computational imaging capabilities, are used for making diagnoses with the help of AI through deep learning. These robots are also used for doing diagnosis procedures like MRIs and X-rays and hence are of great advantage for healthcare workers, as it protects them from harmful radiations used in these procedures [15].

Future perspective

The healthcare segment is globally recognized as one of the most dynamic and biggest industries. It aims to expedite development through modernization and original approaches. Previously this sector was reliant upon manual processes which required more time and were prone to human errors. The latest discoveries in machine learning have brought a revolution in the health sector which aims to create intelligent machines that work and respond like actual persons [8]. Although the application of AI and robotics in the healthcare sector is still in its infant stage, the future seems to be very bright in terms of acceptability and viability [21]. The fields prone to fast adaptability of AI and robotics in healthcare are as follows:

Care for Elderly People

It is predicted that the population of elderly people will double globally by 2050. Socially assistive robot technology may emerge as a solution to this growing demand. The major factors that enhance loneliness among older people living alone are ownership of the house, marital status, bad health, and lack of people to support. A study conducted by Abdi et al. has revealed that the role of social robots is crucial in healthcare of the elderly people [22]. Although many participants of the study were hesitant to accept the significance of robots taking their care, it was quite evident that they were equally apprehensive about having humans as caretakers. Many participants accepted that humanoid robots are programmed with positive human qualities and therefore are more reliable than humans. It can be said that role of robots in taking care of elderly people will prove to be a milestone in the present scenario where the number of elderly people is increasing in India due to improved health services and there is an apparent gap between the demand and supply of trained professionals in hospitals to address the surging need [22].

Mental commit robots are being developed for the therapy of elderly patients in hospitals. These robots are capable of providing a psychological, physiological, and social impact on human beings through physical contact. It was observed that the mood of elderly people improved with this input [23]. Several studies are underway to explore the possibilities of expanding the capabilities of social robots to improve their communication with human beings. The physical appearance of the robot largely influences its acceptability by elderly people. Positive results have been seen in older adults suffering from dementia when they were provided with companion animal robots. Studies demonstrate that companion animal robots of appropriate size, weight, and shape are capable of providing cognitive stimulation to elderly people having dementia [24]. Animal robot like seal PARO developed by Japan's National Institute of Advanced Industrial Science and Technology (AIST) have proven to be quite advantageous for improving the cognitive abilities and sleeping patterns of older adults [25].

Drug Discovery

One of the major areas where the use of AI can prove to be a boon is the field of drug discovery. It takes about 14 years and an average of 2.6 billion dollars for a new drug to reach the market through conventional procedures, whereas the same can be done using AI in a lesser amount of time. Recently in 2015, the outbreak of the Ebola virus in West Africa and some European countries were controlled with the application of AI which helped to discover an appropriate drug in a very meager time and prevented the outbreak from becoming a global pandemic [8]. In addition to this, it has been proven that it takes very little time to conduct clinical trials of newly discovered drugs using AI [8]. AI can also be used to recognize cardiotoxic and non-cardiotoxic drugs of the anticancer group. It is also capable of identifying probable antibiotics from a list of thousands of molecules and can be used as a medium to discover new antibiotics. These algorithms are also being used to identify the molecule with the potential to combat antimicrobial resistance leading to resistance from antibiotics. Studies are underway to explore the role of AI in combating fast-growing antibiotic resistance [26]. 

AI in Diagnosis

Reports say that about 80,000 people die every year due to wrong diagnoses of illnesses. Loads of excessive cases with partial details have led to severe mistakes in the past. As AI is resistant to these errors, it is capable of predicting and diagnosing diseases at a faster pace [27]. The use of AI is extensively explored in the detection of cancer where early detection and prediction are very important. Many companies are using AI-supported tools for diagnosing and detecting different kinds of cancer [28].

Boost in Clinical Trials

Previously the process of clinical trials was very slow and success rates were very poor. Before the year 2000, the success rate of completing the clinical trials via all three stages, for the candidates was only 13.8% [29]. The execution of AI has reduced the cycle time and has also impacted the production cost and outcome in a positive direction. The AI helps in ensuring the continuous flow of clinical trial data and also coding, storing, and managing them. Details of patients saved in the computer can be analyzed and the lessons learned can be used for future trials, thus saving time and cost [30]. It also works efficiently to observe the patients consistently and share the data across different computers. The self-learning capacity of AI enhances the accuracy of the trial and foresees the chances of dropouts [31].

Consultation in Digital Mode

The idea of digital consultation is aimed at lessening hospital visits for minor ailments, which can be cured easily at home with the guidance of a medical professional. Several apps are using AI for collecting information from patients based on a questionnaire and then facilitating the consultation with a medical practitioner [32]. In the future, digital consultation through AI will be the most viable and efficient way for the treatment of common diseases. It would also help people to find good doctors near their houses with the help of AI and internet hospitals.

Remote Patient Monitoring

The concept of remote patient monitoring has evolved very fast with the application of AI sensors and advanced predictive analysis. Apart from personal sensors and devices for monitoring health like glucometers, blood pressure monitors, etc., more advanced systems are now coming up like smart implants and smart prosthetics which are used for post-operative rehabilitation purposes to avoid complications after surgery. Smart implants help in monitoring the patient's conditions such as movements, muscle strength, etc which are important parameters for assessing the rate of recovery. Sensors implanted within the muscles or nerves are quite helpful in providing consistent information about the healing process of the patient.

In recent times many new forms are coming up for patient monitoring, such as digital pills, nanorobots, smart fabrics, etc. These monitoring tools are used for ensuring regular medication, wound management, and management of cardiac diseases by keeping track of patients' emotional, physiological, and mental status [33]. It is calculated that by 2025 the market of AI-based monitoring tools and other wearables will be widely accepted by 50% of the population in developed countries [34]. The initial data and the details during the time of discharge are collected through cell phones having Wi-Fi or Bluetooth. It is further stored in the cloud and constant monitoring is done to avoid complications and readmissions to the hospitals. The review is shared with the patient with recommendations through the internet [35].

AI in Nanotechnology Research

Recent advances have been made in the field of medicine using nanotechnology. AI tools can be successfully merged with nanotechnology to understand the various events happening in the nanosystems. This can help in designing and developing drugs by developing the nanosystems [36]. The field of nanomedicine has grown and continues to develop, numerous approaches have been experimented with successfully to provide several curative instruments in predetermined doses. This advancement has greatly helped in getting efficient results in combination therapy [37].

Prediction of an Epidemic Outbreak

**Figure 2:**
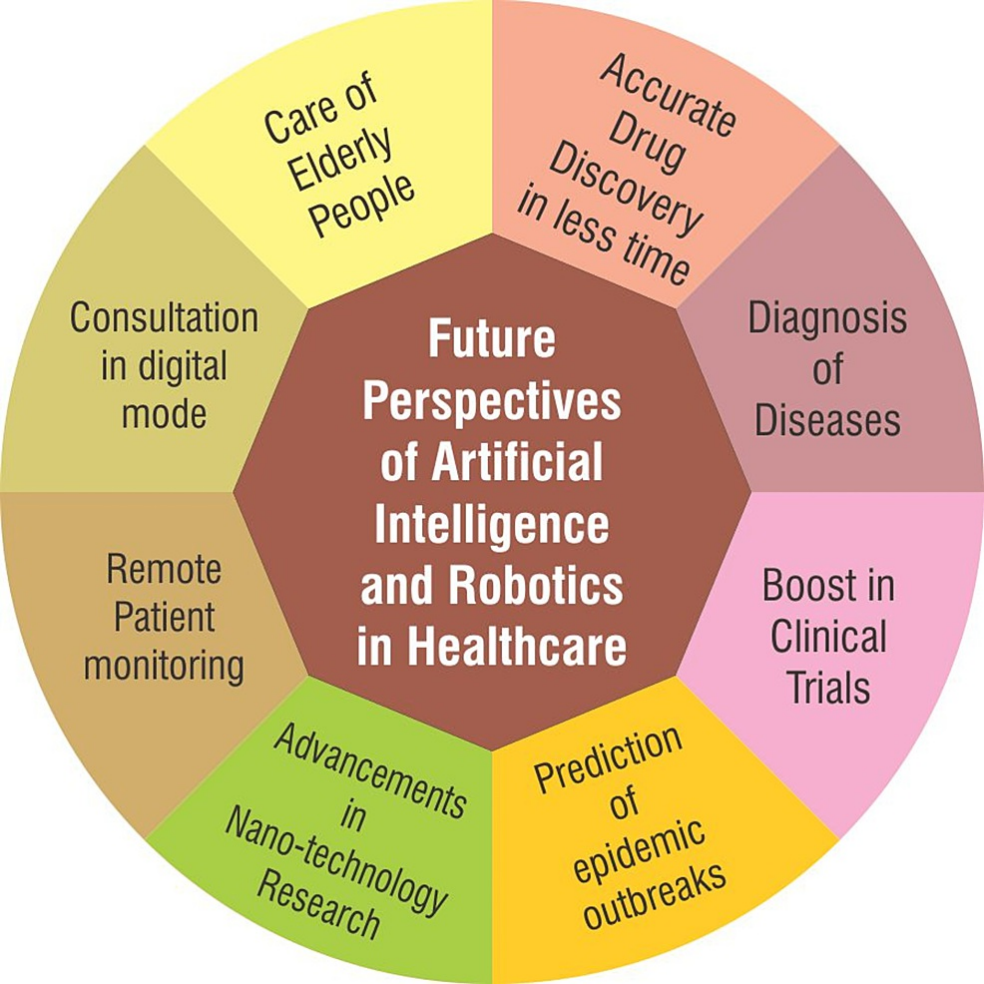
Future perspectives of Artificial Intelligence and Robotics in Healthcare. Image credit: Niyati Deo

One of the most amazing tasks of AI in healthcare is that it is capable of forecasting the outbreak of an epidemic. Although it cannot control or mitigate the outbreak, it can warn us beforehand to make preparations in time. It gathers, analyses and monitors the inflow of data through machine learning or social networking sites to locate the epicenter of the endemic. The calculation is done by generating an algorithm by collecting all the data from the news bulletins in all languages, airline ticketing, and reports related to plant and animal diseases [38]. On 30th December 2019, the AI engine Blue Dot found groups of uncommon pneumonia cases occurring in the wet and dry markets of Wuhan, China, and alerted the government and other stakeholders. This was the first warning signal of the novel COVID-19 pandemic [39]. Figure 2 depicts the various future perspectives of AI and robotics in the field of healthcare.

Barriers to using AI in India

Besides the innumerable benefits of employing robotics in health facilities, there are chances of errors and mechanical failures too. One mechanical breakdown can cost a precious human life. Apparently, there are several disadvantages of robots in the healthcare sector, especially in the Indian scenario.

High cost is the major limitation of introducing robotics in healthcare. In India priority is given to the large burden of contagious diseases like tuberculosis and malaria. The introduction of robotics will be an additional load on the meager budget of the healthcare sector for non-prioritized work. The cost of buying and maintaining the robots is very high. Besides this, the expenditure is very huge for setting up a unit appropriate for robotic operations. 

Another drawback of the present robotic systems used for different healthcare applications is their narrow spectrum for customization. Every patient is different and hence, customization of the healthcare service systems is the need of the hour, for both patients as well as healthcare professionals. Hence, the current healthcare system needs to be more flexible in respect of providing robotic services that can be easily acceptable as per the patient's needs [40]. The use of surgical robots is practically limited to developed countries, advanced research centers, and super specialty hospitals. Practically it is out of reach for patients from a very big section of society in India who actually need it. Expensive robotic interventions are not feasible at the small town and village hospitals where they are actually needed due to excessive workload and lack of health professionals in government-owned health facilities.

Studies related to adverse events in robotic surgery show that several undesirable events were recorded including injuries and deaths due to device fault. Robots are mechanical devices and are susceptible to breakdowns and errors. Shortage of power and lack of other infrastructural facilities do not permit access to the use of robotics universally in the Indian healthcare system. In addition to this, positions of medical professionals at the grass root level are largely vacant and the lack of a trained and skilled workforce for operating and maintaining the robotics and AI system is a challenge. The interconnection between AI and computer programming has a major impact on health and care innovation, where benevolent service delivery systems are increasingly becoming important. These mechanical systems focus on affinity, including the essence of passionate and moral relationships along with therapeutic considerations [12].

Due to its growing popularity, there is also a threat of an increase in irrational demand for robotic surgery in India where the literacy rate and awareness about health are poor. This may lead to hospitals buying robots for commercial publicity and push doctors into unethical use of robotics.

The use of robotics in healthcare also has major medico-legal problems. Like other computers, the surgical robot may also be affected by virus threats and may not adhere to the surgeon's commands, thus leading to a hazardous situation. The government has taken steps to strengthen the medical education system and the delivery of healthcare in rural areas. The introduction of robotics working with mechanical procedures in the healthcare sector in India will possibly deduct the empathy and humanitarian aspect of treatment which is highly appreciated in the Indian scenario where a big percentage of the population is illiterate with low socio-economic status.

Apart from this, there are insufficient laws to address security and privacy issues arising out of data storage through artificial intelligence in the healthcare sector in India [2]. Quality training of the huge and diversified workforce related to the use of AI and robotics in healthcare is another major challenge that needs to be addressed. More and more simulation-based trainings are required to be performed at all levels to enhance the skills of surgeons regarding minimally invasive and robotic colorectal surgery [18]. 

## Conclusions

Although the introduction of robots in healthcare is in its infant stage, it offers a lot of opportunities for medical professionals, especially in the urban setting. The significant role of AI in areas like drug discovery, diagnosis of diseases, digital medical consultations, robotic surgeries, remote patient monitoring and prediction of epidemic outbreaks cannot be denied. The emerging role of robotics in care of elderly people has been recognized and is gradually being accepted by Indian society. In the present scenario it is not possible to think about implementation and monitoring of health services in the absence of AI and robotics. Many new techniques are underway in the use of robotics in the health sector which may be more cost-effective in the future. But the quality of robotic procedures needs to be controlled by establishing a stringent and continuous monitoring system. Use of AI and robotics in healthcare sector in India may prove to be a milestone in improving the present status of healthcare services. It has certainly helped in bridging the gap created by lack of skilled health professionals as well as the huge vacancies of doctors, nurses and paramedical staff. The main challenge is to reach the remote regions of the country with poor infrastructural facilities and lack of advanced technologies. The high cost of using AI and robotics in the healthcare sector stands as the major barrier in the path of reaching the disadvantaged community. Besides this, there are chances of errors and mechanical failures due to improper maintenance arrangements resulting in fatal consequences. The Indian government should support companies to invest in AI and encourage public-private partnership (PPP) in the domain of AI and health. The ethical issues must be addressed by the policy makers to enhance the use of AI and robotics in the healthcare sector. After considering the various facts and practicality, it can be said that the use of robotics in India should be expanded in a phased manner initiating with the reputed and equipped hospitals. It is viable only if used judiciously with a standardized reporting and monitoring system in place. 
